# Transcriptome profiling in susceptible and tolerant rubber tree clones in response to cassiicolin Cas1, a necrotrophic effector from *Corynespora cassiicola*

**DOI:** 10.1371/journal.pone.0254541

**Published:** 2021-07-28

**Authors:** Sébastien Ribeiro, Philippe Label, Dominique Garcia, Pascal Montoro, Valérie Pujade-Renaud

**Affiliations:** 1 Université Clermont Auvergne, Institut National de Recherche pour l’Agriculture, l’Alimentation et l’Environnement, UMR PIAF, Clermont-Ferrand, France; 2 UMR AGAP Institut, Université Montpellier, CIRAD, Institut National de Recherche pour l’Agriculture, l’Alimentation et l’Environnement, Institut Agro, Montpellier, France; 3 CIRAD, UMR AGAP Institut, Montpellier, France; 4 CIRAD, UMR AGAP Institut, Clermont-Ferrand, France; Kunming University of Science and Technology, CHINA

## Abstract

*Corynespora cassiicola*, a fungal plant pathogen with a large host range, causes important damages in rubber tree (*Hevea brasiliensis*), in Asia and Africa. A small secreted protein named cassiicolin was previously identified as a necrotrophic effector required for the virulence of *C*. *cassiicola* in specific rubber tree clones. The objective of this study was to decipher the cassiicolin-mediated molecular mechanisms involved in this compatible interaction. We comparatively analyzed the RNA-Seq transcriptomic profiles of leaves treated or not with the purified cassiicolin Cas1, in two rubber clones: PB260 (susceptible) and RRIM600 (tolerant). The reads were mapped against a synthetic transcriptome composed of all available transcriptomic references from the two clones. Genes differentially expressed in response to cassiicolin Cas1 were identified, in each clone, at two different time-points. After *de novo* annotation of the synthetic transcriptome, we analyzed GO enrichment of the differentially expressed genes in order to elucidate the main functional pathways impacted by cassiicolin. Cassiicolin induced qualitatively similar transcriptional modifications in both the susceptible and the tolerant clones, with a strong negative impact on photosynthesis, and the activation of defense responses *via* redox signaling, production of pathogenesis-related protein, or activation of the secondary metabolism. In the tolerant clone, transcriptional reprogramming occurred earlier but remained moderate. By contrast, the susceptible clone displayed a late but huge transcriptional burst, characterized by massive induction of phosphorylation events and all the features of a hypersensitive response. These results confirm that cassiicolin Cas1 is a necrotrophic effector triggering a hypersensitive response in susceptible rubber clones, in agreement with the necrotrophic-effector-triggered susceptibility model.

## Introduction

*Hevea brasiliensis* is a latex-producing tropical tree exploited for natural rubber production. With 14.3 million tons of natural rubber produced worldwide in 2018 (www.fao.org/faostat/), the rubber tree is a crop of major economic importance. However, a number of cryptogamic diseases threaten this production chain, among which Corynespora Leaf Fall (CLF) disease, caused by the necrotrophic fungus *Corynespora cassiicola* (Berk. & M.A. Curtis) C.T. Wei. Classical leaf symptoms are brown-edged papery lesions of irregular shape, surrounded by a yellow halo. In susceptible rubber clones (i.e. grafted cultivars), the young leaves turn yellow and fall. In mature leaves that escaped leaf fall, blackening of the veins causes typical "fishbone" or "railway track" lesions, considered a signature of the disease. On the most susceptible clones, leaf damages and repeated defoliations result in a significant erosion of latex yields, which may lead the planters to uproot their trees.

A small protein named cassiicolin, secreted by the fungus, was found to play an important role in virulence. Cas1 is the first (and to date, only) cassiicolin isoform functionally characterized. It was extracted from the culture filtrate of a highly aggressive strain (CCP), isolated from diseased rubber trees in the Philippines [1–3]. It is a phytotoxic, cysteine-rich, small secreted glycoprotein (SSP) involved in the early stages of fungal infection. When detached leaves of the susceptible clone PB260 are inoculated with CCP spores, the first CLF disease symptoms appear in 3 to 4 days [1, 4] and are preceded by a peak of cassiicolin *Cas1* gene expression at 1 or 2 days post-inoculation [5]. In the tolerant clone RRIM600, *Cas1* gene expression peaks around 24 hours later compared to PB260. Application of purified cassiicolin Cas1 on PB260 leaves, after local abrasion of the epidermis, induces electrolyte leakages, with a peak around 48 hours after application, concomitant with the first visible leaf symptoms [6].

Besides cassiicolin, other candidate effectors identified *in silico* were found to be transcriptionally regulated 12 to 24 hours after inoculation with CCP, in the susceptible clone PB260 [7]. However, analysis of a mutated CCP strain deleted for the cassiicolin gene *Cas1* demonstrated that Cas1 is required for the successful development of the *C*. *cassiicola* strain CCP in susceptible rubber tree clones, and suggested that it may act as a necrotrophic effector [4]. Other candidate effectors up-regulated upon infection may putatively be involved in saprotrophy in senescing tissue or in necrotrophy, in other susceptible rubber tree clones or other host plants. Cassiicolin toxicity in rubber tree is clearly clone-selective, with polygenic determinism. Indeed, at least two QTL associated with sensitivity to cassiicolin Cas1 were detected in a mapping population derived from the biparental cross PB260 x RRIM600. These QTL were not detected in response to filtrate from the deletion mutant [4, 6].

During biotic stress, a molecular dialogue takes place between host plants and pathogens, which determines the outcome (compatibility or incompatibility) of the interaction. This dialogue begins with the recognition of MAMPs (microbe-associated molecular patterns) or DAMPs (damage-associated molecular pattern) by plant membrane receptors, causing the establishment of a first line of defense known as PTI (pattern-triggered immunity) [8]. Pathogens have evolved specific effector molecules aimed at overcoming this unspecific basal defense line. The perception of these effectors by a specialized intracellular plant receptor triggers a rapid and violent second layer of immune response, leading to local cell death at the point of infection. This hypersensitive response (HR), able to stop the propagation of biotrophic pathogens (which highjack host cell metabolism without killing them), is thus referred to as ETI (effector-triggered immunity) [8]. However, HR seems to be inefficient against, or even beneficial to, necrotrophic pathogens, which are able to survive in such a hostile environment and thrive on dying cells. In this situation, HR seems to confer susceptibility rather than resistance and leads to a compatible interaction. It is thus referred to as NETS (necrotrophic effector-triggered susceptibility) [9, 10].

Cassiicolin Cas1 is an important interactor of CLF disease, required for the virulence of specific *C*. *cassiicola* isolates, such as the highly aggressive CCP, on susceptible rubber tree clones [4]. In this study, we address the question of the molecular mechanisms involved in this compatible interaction. To elucidate the main functional pathways impacted by cassiicolin in rubber tree, we used RNA-Seq technology to comparatively analyze the gene expression profiles of leaves treated or not with purified cassiicolin Cas1, in the sensitive/susceptible clone PB260 and the tolerant clone RRIM600. We first assembled and re-annotated a synthetic transcriptome gathering all available transcriptomic references from the two clones. We then mapped the RNA-Seq reads against the synthetic transcriptome and identified the genes differentially expressed (DEGs) between the Cas1-treated condition and the control condition (water), for each clone. Finally, we performed a gene ontology (GO) enrichment analysis to highlight the major biological functions impacted by the toxin, in the two clones comparatively.

## Material and methods

### Plant material and treatments

The resistant (RRIM600) and susceptible (PB260) rubber clones were cultivated in a greenhouse (Clermont-Ferrand, France) at 27°C for 16 hours (day) and 25°C for 8 hours (night), with 70% relative humidity. For each clone, leaflets were collected at developmental stage C and placed on wet paper, abaxial face up, in 245 × 245 mm Nunc^™^ bioassay dishes (Fisher Scientific, Hampton, NH, USA). They were treated with six drops (10 μl) of either purified cassiicolin Cas1 at 1 ng/μl or sterile water, after local abrasion of the lower epidermis (1 mm^2^), as previously described [6]. Inoculated leaflets were incubated at 26°C in the dark, for 12 or 24 hours before tissue sampling. Three leaf disks (2.2 cm^2^) were sampled from each leaflet, at the abrasion points, and immediately frozen in liquid nitrogen. Four and three biological replicates were performed per treatment at each time point, for PB260 and RRIM600 respectively.

### RNA preparation and sequencing

Total RNAs were extracted from toxin and water-treated leaf disks using CTAB extraction buffer [11], and treated with RNase-free RQ1 DNase (Promega, Madison, WI, USA). The concentration and purity of total RNAs were checked using a NanoDrop ND-1000 spectrophotometer (Thermo Scientific, Wilmington, DE, USA) and by agarose gel migration. RNA sequencing was carried out at the GenoToul GeT-PlaGe Platform (INRA Auzeville, France). Libraries were prepared from 2 μg of total RNA using Illumina TruSeq Stranded mRNA kit (Illumina Inc., San Diego, CA, USA). Paired-end reads (2 x 150 bp) were generated using the Illumina HiSeq3000 sequencing system. The quality of the raw reads was verified using FastQC program [12].

### Cleaning of raw short reads and mapping

RNA-Seq data processing was carried out using our custom pipeline [13] implemented on the GenoToul computing cluster (INRA Auzeville, France). Briefly, 150 bp paired-end short reads were first cleaned: suppression of vector/primer sequences (TagCleaner 0.16 program) [13]; removal of poly-A, poly-T, anonymous nucleotides (N) and reads too short (< 60 bp); filtering of low complexity and decontamination. The Ns were removed to keep only the longest N-free sequence part. Complexity filtering was based on a Lempel-Ziv-Welch compression ratio above 30% [14]. Decontamination was performed by mapping short reads against a database of potential contaminants (bacteria, virus, human, other fungus) using Burrows-Wheeler Alignment (BWA) algorithm [15]. Finally, the cleaned and decontaminated short reads were mapped using BWA (parameterized with 1 nucleotide mismatch) on a *Hevea brasiliensis* so-called synthetic transcriptome (185,685 transcripts) gathering all available transcriptomic references from the two rubber clones studied: PB260 [16] and RRIM600 [17, 18]. A percentage of mapping was calculated for each of the twenty-eight samples as (mapped reads/total reads)x100.

### Differential expression analysis

The count of total mapped reads per transcript and per sample was used as an input dataset to detect differentially expressed genes (DEGs) between toxin-treated (TOX) and abraded control (AC) samples, using DESeq2 package version 1.18.1 [19] in the R environment version 3.4.4. For each clone, the statistical model combined the treatment (TOX and AC), the duration of treatment (12h and 24h) and their interaction. Four sets of two-by-two comparisons were carried out: PB260-TOX-12h *vs* PB260-AC-12h, PB260-TOX-24h *vs* PB260-AC-24h, RRIM600-TOX-12h *vs* RRIM600-AC-12h and RRIM600-TOX-24h *vs* RRIM600-AC-24h, controlled at a False Discovery Rate (FDR) of 0.01, 0.0001, 0.001 and 0.001, respectively, in order to accept no more than four false positives per dataset. The read count distribution was normalized by applying the variance stabilizing transformation (VST) method in order to: perform a principal component analysis (PCA), visualize DEGs and represent the similarity between samples, using plotPCA, plotMA (DESeq2 package) [19] and heatmap.2 (gplots package) [20] respectively. Then, DEGs were filtered to eliminate transcripts with an FDR-adjusted p-value (padj) > 0.001 and those with a log2 fold change value between 1 and -1, in order to maximize the contrast of the biological response. These filtered DEGs were compared between treatment conditions using heatmap.2 based on the log2 fold change values, as described above. Hierarchical clustering was calculated using Euclidean distances and the Ward method. Finally, the unique and common DEGs between rubber tree clones were visualized by a Venn diagram using the VENNY online tool version 2.1 [21].

### Functional annotation of the rubber tree synthetic transcriptome using Blast2GO

The synthetic transcriptome (185,685 transcripts) was re-annotated by running Blast2GO software version 5.2.5 [22]. The blast top-hit annotation (based on e-value) was determined by using a BLASTx analysis against the non-redundant (nr) NCBI database (*Viridiplantae* subset) with default parameters. Transcripts with BLASTx results were mapped and annotated using the corresponding functions of Blast2GO. In the same way, domain/motif information was searched by running InterProScan. All annotations are available in S1 Table.

### GO enrichment analysis

For each clone and each time-point, gene ontology (GO) terms of up-regulated and down-regulated DEGs were assessed separately for enrichment using Fisher’s exact test from Blast2GO software version 5.2.5 [22]. Briefly, each functional annotation subset was compared against the synthetic transcriptome as reference. When available, the thirty most significant enriched GO terms (sorted according to their p-value) were selected, by category (molecular function, biological process and cellular component). The REVIGO program was used to reduce complexity by removing redundant GO terms [23]. The most informative results were visualized as treemaps, with GO loosely related terms grouped in superclusters under a common name (labelled in white squares in the figures).

### Data deposition

The generated RNA-seq data have been deposited in NCBI-SRA database under the project ID PRJNA727474.

The synthetic transcriptome, gathering non redundant, previously published, transcriptomic sequences from PB260 [16] and RRIMM600 [17, 18], is available *via* le following link: https://doi.org/10.6084/m9.figshare.14565426.v1.

## Results

We analyzed the transcriptomic modifications induced by cassiicolin Cas1, virulence effector secreted by *C*. *cassiicola* isolate CCP, in two rubber clones, susceptible (PB260) and tolerant (RRIM600) to CCP respectively.

### RNA-seq metrics

To allow for robust interpretation of the biological data, we checked parameters such as mapping percentages, sample dispersions according to variables and the strength of biological replicate conservation.

A total of 730,063,355 reads were obtained from the 28 samples, averaging 32 million and 18 million reads per library for PB260 and RRIM600, respectively. After cleaning off the low-quality reads, the data were mapped on the 185,685 transcripts of the synthetic rubber tree transcriptome. On average, 96% of the cleaned reads were mapped, with mapping percentages ranging from 89 to 98 depending on the samples. Total reads, mapped reads and percentage of mapping per sample are given in S2 Table.

A principal component analysis (PCA) of the normalized read counts was performed for each rubber clone to explore the treatment (TOX and AC) and duration of treatment (12h and 24h) effects on the variability between samples (S1 Fig). For PB260 (S1A Fig), the first component (PCA1), mostly contributed by the duration of the toxin treatment, explained 93% of the total variance and discriminated the 24 hours toxin-treated samples from all others. It was noticed that the R4 replicate was not perfectly grouped with the other three, whether at 12 or 24 hpt (hours post-treatment). For RRIM600 (S1B Fig), the first two components accounted for 79% of the total variance, discriminating cassiicolin-treated leaves from control leaves on the first axis (PCA1, 62%), and the treatment duration (12 or 24 hours) among cassiicolin-treated leaves on the second axis (PCA2, 17%). Overall, PCA analysis evidenced a significant effect of cassiicolin application on gene expression, at both 12 and 24 hpt for RRIM600 but at 24 hpt only for PB260.

We analyzed the sample-to-sample distances for each rubber clone to further explore the similarities and dissimilarities between replicates (S2 and S3 Figs). Except for the condition PB260-12h, each heatmap clearly separated two major clusters corresponding to the TOX and AC samples respectively, with a positive correlation among replicates within the same condition.

### Analysis of the differentially expressed genes (DEGs)

To identify genes differentially expressed in response to cassiicolin, we analyzed the changes in transcript amounts between the cassiicolin-treated and -untreated rubber leaves, for each clone at each time-point. Since leaves were locally abraded in both treated and untreated conditions, DEGs identified here can be considered as significantly associated to the cassiicolin response. However, we cannot exclude that transcripts regulated by both wounding and cassiicolin may be missed.

**Fig 1:**
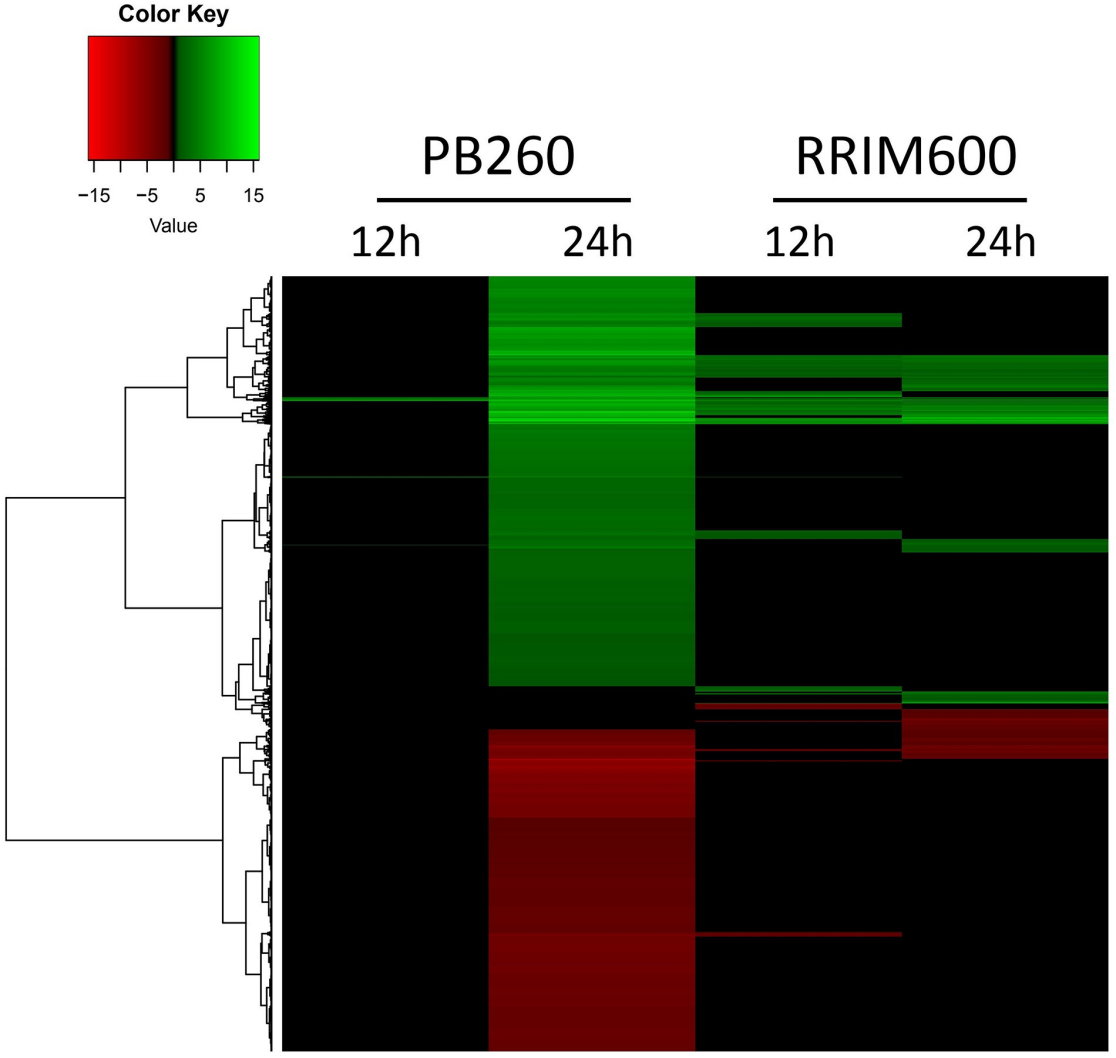
Heatmap of DEGs 12h and 24h after treatment with purified cassiicolin, in PB260 and RRIM600. Differentially expressed genes (DEGs) between toxin-treated and abraded control leaves were identified in the four conditions: PB260-12h, PB260-24h, RRIM600-12h and RRIM600-24h. The heatmap was generated with log2 fold change values using the heatmap.2 function from the gplots package. The dendrogram represents gene clustering, using the Euclidian method. The green and red colors indicate up-regulation (log2 fold change > 1) and down-regulation (log2 fold change < -1), respectively. Black color denotes a non-differential expression (log2 fold change strictly between -1 and 1). Each condition represents the mean of 3 (RRIM600) or 4 (PB260) biological replicates.

Both susceptible and tolerant clones reacted to the cassiicolin treatment by transcriptional modifications, although with important differences in terms of timing or response intensity (Table 1 and Fig 1). Twelve hours after toxin application on leaves of the susceptible clone PB260, only 162 transcripts were differentially expressed (158 up-regulated and 4 down-regulated), relatively to the abraded toxin-free control. After 24 hours, by contrast, 11,802 genes were up-regulated and 9,290 down-regulated, suggesting massive transcriptional reprogramming. In the RRIM600 tolerant clone, the influence of treatment duration was less marked, with 2,775 DEGs at 12 hours (2,349 up-regulated and 432 down-regulated) against 3,952 DEGs at 24 hours (2,529 up-regulated and 1,423 down-regulated). Overall, PB260 reacted later but stronger while RRIM600 reacted earlier but with moderate response intensity (in number of DEGs) at both time-points. The detailed list of all DEGs is given in S3 Table.

**Table 1:** Numbers of DEGs 12 and 24 hours after cassiicolin application on PB260 and RRIM600 rubber leaves.

	PB260 (susceptible)				RRIM600 (tolerant)			
	12h		24h		12h		24h	
	Up	Down	Up	Down	Up	Down	Up	Down
Number of regulated genes	158	4	11,802	9,290	2,349	432	2,529	1,423
Mean of log2fc	3.65	-1.25	3.80	-2.77	2.69	-1.83	3.24	-2.05
Maximum of log2fc	9.15	-1.03	15.21	-1.01	10.96	-1.01	13.07	-1.01
Minimum of log2fc	1.02	-1.46	1.01	-9.55	1.01	-7.61	1.01	-8.92
**Total**	**162**		**21,092**		**2,775**		**3,952**	
FDR	0.01		0.0001		0.001		0.001	
Expected false positives	1.6		2.1		2.8		3.9	

For each condition (PB260-12h, PB260-24h, RRIM600-12h and RRIM600-24h), data were divided into up- and down-regulated genes. The mean, the maximum and the minimum expression values were given as log2 fold change (log2fc). The FDR (false discovery rate) was adapted to the number of DEGs in each condition in order to accept no more than 4 false positives per dataset, after correction (with FDR adjusted p-values)

The DEG distribution patterns are illustrated in the MA plots (S4 Fig). All four conditions showed a higher number of up-regulated genes compared to down-regulated genes. In PB260, the number of DEGs (in red) increased drastically at 24 hpt compared to 12 hpt, notably DEGs with both a high mean of normalized counts and high log2 fold changes. In RRIM600 at 12 hpt, more DEGs were observed than in PB260 at the same time-point. However, the changes between 12 and 24 hpt were not as strong.

**Fig 2:**
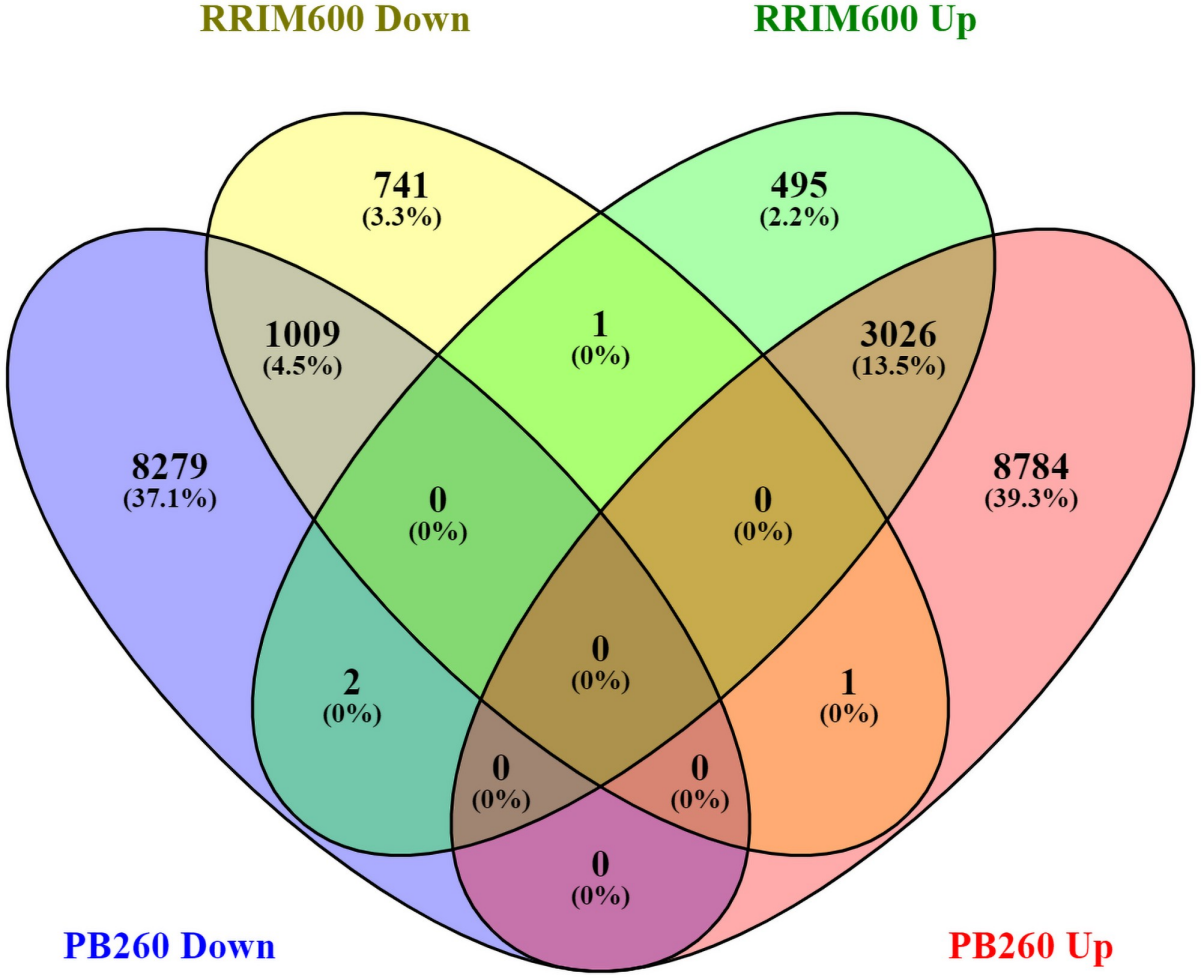
Venn diagram displaying the overlaps between PB260 and RRIM600 DEGs. The DEG lists were extracted from S3 Table. For this analysis, transcripts expressed at 12 hpt and 24 hpt were combined for each clone. The up-regulated (green for RRIM600 and red for PB260) and down-regulated (yellow for RRIM600 and blue for PB260) genes were counted separately. The diagram was drawn online using the VENNY tool (version 2.1).

The number of up- and down-regulated genes of each clone was visualized in a Venn diagram (Fig 2). The duration of treatment was not taken into account (for each clone, the 12 and 24 hpt DEGs were combined). Overall PB260 and RRIM600 shared 4,038 DEGs, representing 19% of all PB260 DEGs and 60% of all RRIM600 DEGs. Among those, 1,009 were down-regulated, (representing 5% of all PB260 DEGs and 15% of all RRIM600 DEGs) and 3,026 up-regulated (14% of all PB260 DEGs and 45% of all RRIM600 DEGs). Only three transcripts showed opposite expression profiles between the two clones. Two (CL7065Contig1_PB260 and CL1Contig20508_PB260) were up-regulated in RRIM600 and down-regulated in PB260, and one (Hb_076771_010) was down-regulated in RRIM600 and up-regulated in PB260. As shown in S3 Table, CL7065Contig1_PB260 is homologous to a LRR receptor-like serine/threonine-protein kinase; CL1Contig20508_PB260 is homologous to a 3-ketoacyl-CoA synthase; and Hb_076771_010 matched with putative disease resistance RGA3 proteins from rubber tree (clone Reyan7-33-97), although with 95% identity only. Surprisingly, transcript CL1Contig19300_PB260 appeared both down- and up-regulated in RRIM600. This is explained by the fact that, for each clone, the 12h and 24h DEGs were combined. In fact, this transcript is down-regulated at 12 hpt and up-regulated at 24 hpt.

### Annotation of DEGs using Blast2GO

In order to provide recent and comprehensive information about the putative role of DEGs in the response to cassiicolin in PB260 and RRIM600 leaves, the synthetic transcriptome, gathering all available references for these two clones, was re-annotated using Blast2GO software. As show in Table 2, 43% of the synthetic transcriptome was initially annotated with functional BLASTx results (excluding ‘hypothetical’ or ‘uncharacterized’ proteins) or GO terms. After *de novo* annotation of the synthetic transcriptome, the percentages of annotated transcripts increased to 71% for the functional blastx annotations, and to 67% for the GO annotations. The complete *de novo* annotation of the synthetic transcriptome is available in S1 Table. We used this updated resource for the gene ontology enrichment analysis.

**Table 2:** Comparative metrics of functional annotations in published transcriptome references and our synthetic assembly.

	PB260	RRIM600		Synthetic transcriptome	
	Duan *et al*. (2013)	Salgado *et al*. (2014)	Makita *et al*. (2018)	Before re-annotation	After re-annotation
Total transcripts	86,941	34,572	84,443	185,685*	185,685*
Transcripts with blastx annotation	72,911 (83%)	21,976 (63%)	64,585 (76%)	142,977 (77%)	158 757 (85%)
Transcripts without ‘hypothetical’ and ‘uncharacterized’ proteins	29,165 (33%)	20,054 (58%)	44,985 (53%)	85,415 (46%)	131,190 (71%)
Transcripts with GO annotation	30,343 (35%)	21,976 (63%)	35,247 (42%)	79,844 (43%)	123,683 (67%)

The synthetic transcriptome assembles one reference transcriptome from PB260 and two from RRIM600. It was re-annotated using Blas2GO software. For each transcriptome, the number of transcripts with blastx annotation (with or without ‘hypothetical’ and ‘uncharacterized’ proteins) and with GO annotation, are specified. The percentages of annotated transcripts (in parentheses) are related to the total number of transcripts.

*The size of synthetic transcriptome is lower than the sum of three original reference transcriptomes because redundant (identical) transcripts have been deleted. The number of transcripts in this case reflects the presence of allelic forms originating from the different genotypes, as well as differentially spliced forms. However, it can also be inflated by assembly errors in the original reference transcriptomes.

### Functional classification of DEGs by GO enrichment

**Fig 3:**
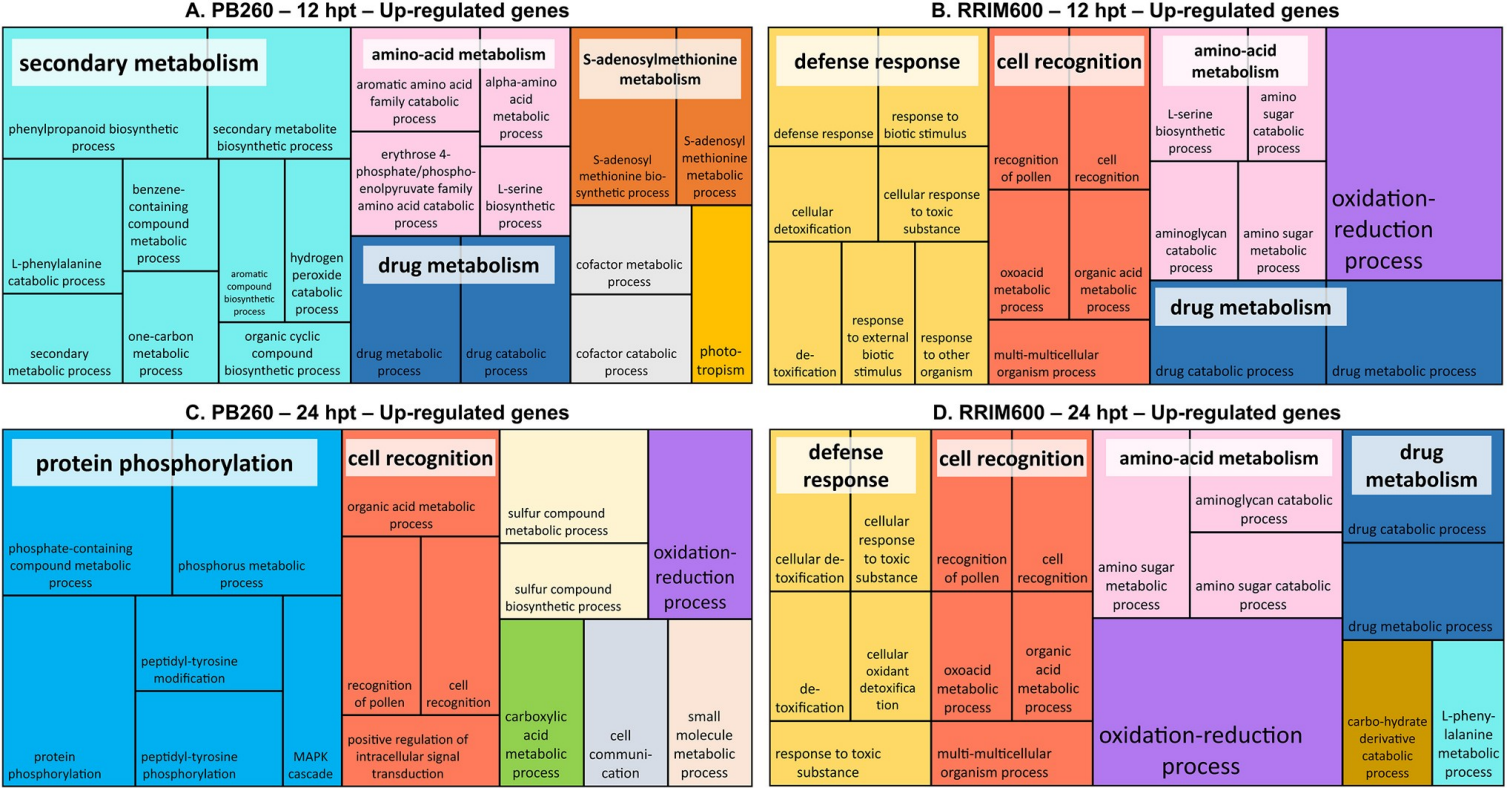
REVIGO treemap representation summarizing GO biological process categories over-represented among PB260 (A and C) and RRIM600 (B and D) up-regulated genes. Treemaps were performed separately for 12 hpt (A and B) and 24 hpt (C and D). They were built using the percentages of genes annotated with the non-redundant GO terms from S4 Table (REVIGO treemap % column). Each rectangle represents a significant GO term, sometimes joined into colorized superclusters of loosely related terms, whose names are labelled in white squares. The size of the rectangles is proportional to the p-value given by the Blast2GO analysis (i.e. the larger the rectangle, the more significant the GO-term).

**Fig 4:**
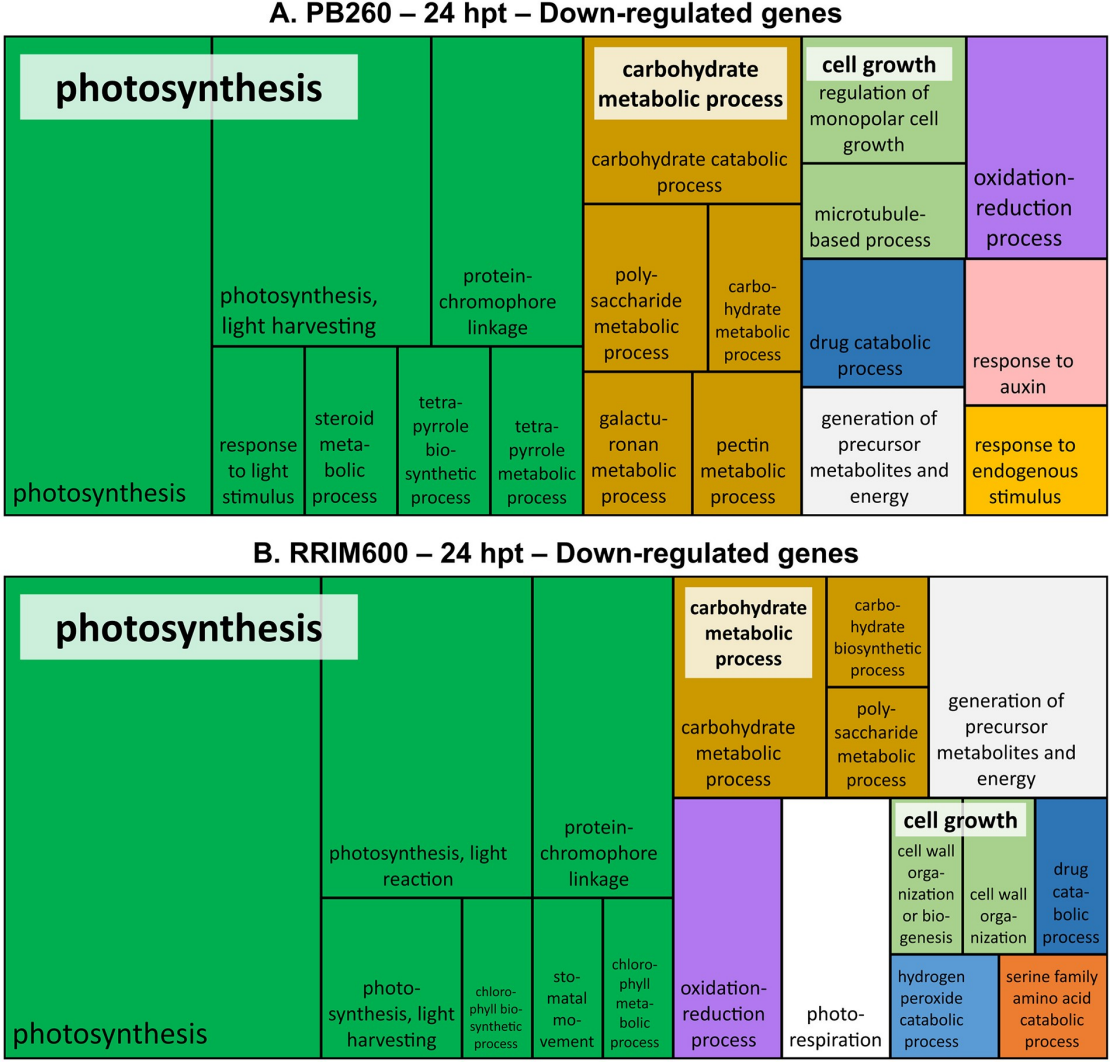
REVIGO treemap representation summarizing GO biological process categories over-represented from PB260 (A) and RRIM600 (B) down-regulated genes. Treemaps were performed only for 24 hpt DEGs. They were built using the percentages of genes annotated with the non-redundant GO terms from S4 Table (REVIGO treemap % column). Each rectangle represents a significant GO term, sometimes joined into colorized superclusters of loosely related terms, whose names are labelled in white squares. The size of the rectangles is proportional to the p-value given by the Blast2GO analysis (i.e. the larger the rectangle, the more significant the GO-term).

To elucidate the functional significance of the transcriptional response induced by cassiicolin in rubber tree leaves, we performed a GO enrichment analysis using Blast2GO software. For each clone (PB260 and RRIM600) at each time-point (12 and 24 hpt), up- and down-regulated genes were analyzed separately (S4 Table). Treemaps in Figs 3 and 4 summarize the GO terms enrichment in the biological process (BP) category, for the up- and down-regulated genes respectively. The same analysis performed in the molecular function (MF) was presented as S5 and S6 Figs.

Among the up-regulated genes, GO enrichment in the BP category (Fig 3) revealed that both clones share several superclusters, although with time-frame shifts. The ‘cell recognition’ and ‘oxidation-reduction processes’ superclusters appeared earlier in RRIM600 (panel B) than in PB260 (panel C). They are the only two superclusters shared by both clones at 24 hpt (panel C and D). The ‘amino-acid metabolism’ and ‘drug metabolism’ superclusters are shared by the two clones at 12 hpt (panels A and B), however at 24 hpt they were observed in RRIM600 only (panel D). The ‘secondary metabolism’ supercluster is especially important among PB260 enriched GO terms at 12 hpt (panel A) while less represented and appearing later in RRIM600 (panel D).

Analysis of the up-regulated genes also evidenced clone-specific responses, such as the highly represented ‘protein phosphorylation’ supercluster, specific to PB260, at 24 hpt (panel C). Several smaller clusters are also specific to PB260: ‘S-adenosylmethionine metabolism’, ‘cofactor metabolic or catabolic processes’, ‘phototropism’ at 12 hpt (panel A); ‘sulfur compound metabolic processes’, carboxylic acid metabolic processes’, and ‘cell communication’ at 24 hpt (panel C). Concerning RRIM600, the main clone-specific supercluster is related to ‘defense responses’, and is found at both 12 and 24 hpt (panels B and D). A smaller cluster specific to RRIM600 is related to the ‘carbohydrate derivative catabolic process’ and found at 24 hpt only (panel D).

At 12 hpt, the number of down-regulated genes in P260 was too low to perform GO enrichment; and in RRIM600, only few GO terms were found enriched (S4 Table). Therefore, only the 24 hpt profiles were presented for the down-regulated genes in the BP category (Fig 4). For each clone, the larger supercluster was found related to ‘photosynthesis’. Five other clusters (carbohydrate metabolic process, cell growth, oxidation-reduction process, drug catabolic process and generation of precursor metabolites and energy) were also common to both clones. While GO terms ‘response to auxin’ and ‘response to endogenous stimulus’ were specific to PB260 (Fig 4A), ‘photorespiration’, ‘hydrogen peroxide catabolic process’ and ‘serine family amino acid catabolic process’ were specific to RRIM600 (Fig 4B).

GO enrichment analysis based on the MF and CC categories (S5 and S6 Figs and S4 Table) confirmed some of the BP-based observations. For example, the ‘oxidoreductase activity’ clusters, highly enriched in RRIM600 but less represented in PB260, are consistent with the ‘oxidation-reduction processes’ profile observed in the BP category. Surprisingly, enrichment in ‘oxidoreductase activities’ in PB260 was detected sooner (12 hpt) in the MF category than in the BP category. The large ‘lyase activity’ supercluster in the MF category supports the corresponding ‘secondary metabolism’ supercluster in the BP category, with a similar profile. GO terms related to ‘transferase activity’, specific to PB260, are enriched at both 12 and 24 hpt (S5 Fig), but only those appearing at 24 hpt seem to be related to phosphorylation, in agreement with the analysis results in the BP category (Fig 3C). The clusters related to ‘chitinase activity’ might explain at the least in part the ‘defense response’ supercluster, specific to RRIM600 in the BP-based analysis. The MF-based analysis stresses other interesting features such as the ‘DNA/nucleotide-binding’ functions, which seem to be more highly represented in PB260 enriched GO terms compared to RRIM600. Overall, many clusters in the MF category were shared by the two clones, although they appeared later (24 hpt) in PB260 than in RRIM600 (24 hpt).

The enrichment profiles of the down-regulated genes in the MF category (S6 Fig) confirm the significant down-regulation of functions related to photosynthesis (chlorophyll binding). As additional evidence GO terms related to the ‘chloroplast’, in the CC category, are also significantly enriched at 24 hpt in both clones (S4 Table).

Overall, one striking result (whether in the PB, MF or CC category) is that the enrichment profiles characterizing RRIM600 transcriptomic response to cassiicolin are similar at 12 and 24 hpt, while in PB260, they vary considerably between the two time points. However, this result should be viewed with caution considering the small number of DEGs at 12 hpt for PB260.

### Immune receptors transcriptionally regulated in response to Cas1

In this study, we have placed special emphasis on genes encoding putative immune receptors, which are key players of the plant immune system. They are classified in two groups, based on their structure and function: PRRs (pattern recognition receptors) are membrane receptors triggering PTI; NLRs (NBS-LRR receptors) are intracellular receptors triggering ETI or NETS. In our study, DEGs identified as putative PRRs or NLRs based on a key word search and data from the literature, were extracted from the different lists of the S3 Table. They are provided in Table 3.

**Table 3:** List of putative PRR- and NLR-encoding genes differentially expressed after cassiicolin treatment on PB260 and RRIM600 leaves.

	Blast identification	Reference	PB260-12h		PB260-24h		RRIM600-12h		RRIM600-24h	
			Up	Down	Up	Down	Up	Down	Up	Down
PRR	**RK-PRR family** (674 different genes)									
	LRR receptor-like serine/threonine-protein kinase	3,648	0	0	164	127	40	7	46	23
	G-type lectin S-receptor-like serine/threonine-protein kinase	962	1	0	150	7	49	2	56	1
	L-type lectin-domain containing receptor kinase	276	0	0	35	11	17	0	12	0
	wall-associated receptor kinase (WAK)	195	1	0	79	7	24	0	16	3
	cysteine-rich receptor-like protein kinase (CRK)	125	1	0	38	3	13	0	7	0
	lysM domain receptor-like kinase	65	0	0	24	4	3	0	4	2
	**RLP-PRR family** (54 different genes)									
	leucine-rich repeat protein	332	0	0	30	10	2	1	3	3
	LysM domain-containing protein	24	0	0	6	3	2	0	1	0
	**Total PRRs**	**5,627**	**3**	**0**	**526**	**172**	**150**	**10**	**145**	**32**
NLR	**TIR-NLR family** (143 different genes)									
	TMV resistance protein N	1,514	0	0	67	8	7	0	4	1
	disease resistance protein TAO1	73	0	0	16	0	1	0	0	0
	disease resistance protein RPS6	57	0	0	3	0	0	0	0	0
	disease resistance protein RML1B	52	0	0	36	0	2	0	0	0
	disease resistance protein RML1A-like	25	0	0	1	1	0	0	0	0
	disease resistance-like protein DSC1	22	0	0	2	1	0	0	0	0
	disease resistance-like protein CSA1	8	0	0	5	0	1	0	1	0
	**CC-NLR family** (51 different genes)									
	disease resistance RPP13	328	0	0	14	12	0	0	0	1
	disease resistance protein RPM1	131	0	0	1	14	0	0	0	1
	disease resistance protein RPP8	29	0	0	5	1	3	0	1	0
	disease resistance protein RPS2	29	0	0	1	0	0	0	0	0
	**Unknown family** (113 different genes)									
	disease resistance protein RGA3	531	0	0	19	11	0	1	0	7
	disease resistance protein RGA4	110	0	0	2	1	0	0	0	1
	disease resistance protein RGA2-like	76	0	0	4	2	0	0	0	0
	disease resistance protein RGA1	56	0	0	5	0	0	0	0	1
	Others	516	0	0	33	24	2	0	5	4
	**Total NLRs**	**3,557**	**0**	**0**	**214**	**75**	**16**	**1**	**11**	**16**

Genes encoding PRR and NLR proteins were listed by combining data from the literature and a keyword search in synthetic transcriptome annotation (S3 Table, blast identification column). PRR were classified into receptor kinase (RK-PRR) and receptor-like protein (RLP-PRR) families, depending on the presence/absence of a C-terminal kinase domain. Several sub-categories were determined according to the N-terminal domains. NLR were classified based on their N-terminal domain into TIR-NLR (containing a toll/interleukin domain) and CC-NBS-LRR (containing Coil-coiled domain). RGA (resistance gene analogs) are yet unclassified NLRs. The number of DEGs was given for the complete reference transcriptome (reference) and for each condition (PB260-12h, PB260-24h, RRIM600-12h and RRIM600-24h), with up- and down-regulated genes indicated separately.

#### PRRs

Out of the 5,627 putative PRR-encoding genes from the reference synthetic transcriptome, 728 were differentially regulated upon cassiicolin Cas1 treatment, all conditions combined (PB260-12h, PB260-24h, RRIM600-12h and RRIM600-24h). In RRIM600, the number of up-regulated PRR transcripts was similar at both time points (150 at 12 hpt and 145 at 24 hpt). By contrast, in PB260, only three PRR transcripts were up-regulated at 12 hpt, against 526 at 24 hpt. Qualitatively, the patterns of differentially expressed PRRs were quite similar between the two clones. All categories were represented among the up-regulated PRRs, with LRR receptor-like serine/threonine-protein kinases and G-type lectin S-receptor-like serine/threonine-protein kinases representing around 30% each (of all up-regulated PRRs). The number of down-regulated PRRs was lower, compared to those up-regulated, but their pattern was similar: the contrast between the two time points was important in PB260 (0 and 172 down-regulated PRRs respectively, at 12 and 24 hpt) but not in RRIM600 (10 and 32 respectively); all categories were represented in PB260-24h but fewer in RRIM600; the LRR receptor-like serine/threonine-protein kinase was the most highly represented (> 70%). Among all PRRs, the highest log2 fold change ultimate value was found for an LRR receptor-like serine/threonine-protein kinase (Hb_000061_360, S3 Table), in both clones, ranging up to 11.37 for PB260-24h, and 9.54 for RRIM600-24h.

#### NLRs

Out of the 3,557 putative NLR-encoding genes from the reference synthetic transcriptome, 307 were differentially regulated upon cassiicolin Cas1 treatment, all conditions combined. In PB260, no NLR gene was found differentially expressed at 12 hpt, while at 24 hpt, 214 were up-regulated and 75 down-regulated. In RRIM600, comparatively, transcriptional reprogramming of NLR genes occurred earlier, with 16 up-regulated and 1 down-regulated at 12 hpt, but it remained moderate, although the number of down-regulated NLRs increased slightly (up to 16 at 24 hpt) Qualitatively all categories were represented among the up-regulated NLRs in PB260, and mostly TIR-NLRs (such as TMV resistance protein N and RML-1B). Among the CC-NLR, the RPM1 category displayed notably more down- than up-regulated transcripts (14 and 1 respectively). Among all NLRs, the highest e-values and log2 fold change ultimate values were found for induced NLRs in PB260-24h, ranging up to 8E-67 (log2 fold change 6,04) for disease resistance protein RPP8 (Hb_002656_020, S3 Table).

## Discussion

CLF disease is responsible for important economic losses in rubber plantations, due to the progressive erosion of latex yields as a consequence of repeated leaf fall. The selection of new clones with better tolerance to the fungus seems to be the best strategy for controling the disease. We previously demonstrated that Cassiicolin Cas1, a small phytotoxic protein secreted by *C*. *cassiicola* virulent strain CCP, was required for the virulence of CPP in a range of susceptible clones [4]. By phenotyping the progeny of a PB260 x RRIM600 family with fungal filtrates or with purified cassiicolin, we identified QTL specifically associated with sensitivity to Cas1 [4, 6]. In this study, we describe the transcriptomic response of these two parental clones, chosen for their contrasted sensitivity to the toxin (Cas1) as well as contrasted susceptibility to the fungus (CCP), with the objective to shed light on cassiicolin-induced toxicity mechanisms in rubber tree. A previous study by Roy and colleagues [24] described the transcriptional modifications induced after inoculation of the *C*. *cassiicola* strain Cc 102 on two rubber clones, RRI105 (susceptible) and GT1 (resistant). We have previously shown that each *C*. *cassiicola* strain can express a large set of putative effectors [7], each expected to interact with a specific plant target, which makes clonal comparisons of transcriptomic responses rather complex. The study we describe here is the first transcriptomic analysis in rubber tree involving an isolated effector purified to homogeneity from a *C*. *cassiicola* culture filtrate.

### Pathways impacted by cassiicolin Cas1 in PB260 and/or RRIM600 clones

The reliability of statistical analyses in transcriptomic analyses increases with the number of independent biological replicates [25, 26]. In our study, the three biological replicates for the RRIM600 clone showed good repeatability. However, for PB260, one of the four replicates did not group perfectly with the other three (S1–S3 Figs). Since no technical explanation was likely to explain this variability, we assumed that it was of biological origin but are unable to explain it. To verify the impact of this variability on the interpretation of the results, we repeated the analyses after removing the divergent replicate (R4). The results obtained being similar, we chose to keep the four replicates to take biological variability into account in the interpretation of the results.

Under these conditions, sixty percent of the genes differentially expressed in RRIM600 were also found differentially expressed in PB260 (Fig 2). By contrast, PB260 DEGs were in the majority specific to this clone. GO enrichment analysis highlighted the metabolic pathways mostly impacted by cassiicolin Cas1, common or specific to each clone (Figs 3 and 4, S5 and S6 Figs). The overall organization of the superclusters was fairly well-conserved between RRIM600-12h, RRIM600-24h, and PB260-24h. The discrepancy of PB260-12h might be due to the much lower number of DEGs in this condition. Transcripts putatively involved both in the wound and cassiicolin responses may remain undetected, especially at the early time points, which could explain the low number of DEGs in PB260-12hpt. As discussed below, GO terms such as ‘photosynthesis’, ‘oxidation-reduction, ‘cell recognition’, ‘amino-acid metabolism’, ‘drug metabolism’, ‘carbohydrate metabolism’, ‘cell growth’, and ‘generation of precursor metabolites and energy’ were enriched in both clones. Other GO terms were more specifically enriched in one or the other clone. This was the case for the superclusters ‘secondary metabolism’ and ‘protein phosphorylation’, highly represented in PB260 but missing or scarcely represented in RRIM600.

#### Photosynthesis, carbohydrate metabolism and cell growth

In both clones, down-regulated genes associated with the chloroplast and the photosynthesis processes (Fig 4 and S4 Table) were significantly enriched. Genes coding for chlorophyll a-b binding protein, ferredoxin and photosystem I and II were the most highly represented (S3 Table), in agreement with Pandelova and colleagues [27] in the *Pyrenophora tritici-repentis* / wheat pathosystem. Collapse of the photosynthetic machinery is frequently observed during biotic interactions [28–32]. It is presumed that the plant mobilizes resources for immediate defense needs, to the detriment of photosynthesis [33] Down-regulation of genes associated with sugar metabolism or cell growth might be a direct consequence of photosynthesis collapse. In our study, these phenomena, occurring in both clones and rather late on (24 h), cannot explain the difference in sensitivity between the two clones.

#### Oxidation-reduction processes

The GO term ’oxidation-reduction process’ (GO:0055114) is one of the most significantly enriched in our study (S4 Table). Oxidation-reduction processes are involved in many physiological phenomena and are essential for the regulation of cell functions. It is therefore not surprising that this GO term (GO:0055114) is enriched in both clones, and covers both up- and down-regulated transcripts. Both DEGs involved in the induction of reactive oxygen species (ROS) or in their detoxification are found under this term. The production of ROS is one of the earliest cellular responses following successful pathogen recognition and activation of plant defenses [34]. In our study, oxidases such as ‘reticuline oxidase’ and the ‘respiratory burst oxidase’, involved in the generation of extracellular H_2_0_2_ [35] were strongly up-regulated in both clones, with log2 fold change values of up to 13.33 in PB260 and 11.77 in RRIM600 (S3 Table).

Enzymes like superoxide dismutase, catalase and peroxidases play key roles in cellular ROS detoxification [36–38]. In our study, peroxidases were found among DEGs (both up and down-regulated) with high log2 fold change ultimate values (up to 13.25), in similar proportions in both clones (S3 Table). SOD and catalases were found down-regulated in both clones. Overall, no major qualitative difference in the pattern of DEGs associated with oxidative stress was observed between the two clones, although the earlier response in RRIM600 may contribute to better tolerance. It seems that to the fungal effector cassiicolin elicits oxidative stress in both clones, probably as part of basal defense mechanisms aiming at stopping the development of pathogens. In a similar study conducted on tobacco infected by a nematode, genes encoding proteins of the antioxidant system were found differentially expressed in both the resistant and the susceptible genotypes [39].

#### Drug and amino-acid metabolisms

The enriched superclusters ‘drug metabolism’ and ‘amino-acid metabolism’ (Fig 3 and S3 Table) include DEGs encoding chitinases. These enzymes are pathogenesis-related (PR) proteins produced during the PAMP reaction, that contribute to plant protection by degrading the fungal wall chitin [40, 41]. In our study, chitinases were found up-regulated in both clones in response to Cas1. Although GO terms associated with ‘drug metabolism’ or ‘amino-acid metabolism’ were not found enriched in PB260-24h, DEGs encoding chitinases, both up- and down-regulated, were also identified in this condition, as in RRIM600-24h.

#### Cell recognition

The enriched supercluster ‘cell recognition’ was found in PB260-24h and in RRIM600 at both time points (Fig 3). It mostly comprises up-regulated DEGs encoding PRR membrane receptors (S3 Table). PRRs are involved in the perception of danger signals (MAMPs and DAMPs), leading to the activation of unspecific basal defenses known as PTI (pattern-triggered immunity). This suggests that cassiicolin activated PTI in both clones, as further discussed in the second part of this section.

#### Secondary metabolism

DEGs associated with the ‘secondary metabolism’, were found highly represented among the up-regulated DEGs in PB260-12h. They mostly encode proteins of the phenylpropanoid pathway, which generates secondary metabolites such as lignins, flavonoids or phytoalexins involved in plant defense against pathogens [42]. However, genes encoding phenylalanine ammonia-lyase (PAL), 4-coumarate:CoA ligase (4CL), cinnamoyl-CoA reductase (CCR) or caffeoyl-CoA O-methyltransferase (CCoAOMT), were also found among the up-regulated DEGs in RRIM600 at the same time point (S3 Table), suggesting that this pathway is likely activated early in both clones, as part of the PTI response. It was probably found over-represented in PB260-12h owing to the low number of DEGs (only 162, Table 1), while in RRIM600, where more genes where impacted, other GO terms were more significantly enriched and masked this pathway.

#### Protein phosphorylation

Terms associated with ‘protein phosphorylation’ were strikingly enriched among the up-regulated DEGs in PB260, at 24h (Fig 3 and S4 Table). The recognition of pathogen effectors by plant receptors activates phosphorylation cascades involving MAPKs (mitogen-associated protein kinases) that modulate the expression of genes leading to defense [43]. MAPKs control for example hormonal synthesis and/or signalization, activation of defense-related genes, synthesis of anti-microbes metabolites, stomatal closure or cell death caused by the hypersensitive response (HR). In our study, MAPKs and other kinases such as wall-associated receptor kinases (WAKs) or calcium-dependent protein kinases were found up-regulated in both clones (S3 Table). Overall, the ‘protein phosphorylation’ process was not found significantly enriched in RRIM600, whether at 12 or 24 hpt. In PB260, considering the huge number of DEGs at 24 hpt, this enrichment is highly significant and probably explains the transcriptional burst observed (Fig 1).

#### Defense response

GO terms related to defense responses were highly enriched in RRIM600 but not in PB260 (Fig 3 and S3 Table). In RRIM600, DEGs classified in the ’defense response’ supercluster were over-expressed at both 12 and 24 hours after cassiicolin Cas1 treatment. Among those are genes encoding lignin-forming anionic peroxidase-like, peroxidases, respiratory burst oxidases and major allergen Pru. The three former enzymes are involved in ROS production and detoxication previously discussed in the ‘oxidative stress’ section. It is not surprising to have the same gene family in two superclusters since the term ’oxidation-reduction process’ is placed very high in the gene ontology hierarchy. The major allergen Pru belongs to the PR-10 family of pathogenesis-related proteins produced by plants when attacked by a pathogen [44]. Some PR-10 proteins were shown to have a direct and selective antifungal effect [45]. In our study, DEGs encoding PR-10, as well as peroxidases, could also be found in PB260 at 24 hpt, but overall, the ‘defense response’ terms were not among the 30 most significantly enriched in the susceptible clone.

Overall, the most striking difference between the two clones, in terms of pathways modified by cassiicolin, is the huge amplification of the phosphorylation cascades in susceptible PB260, which both explains and reflects the frantic transcriptional burst in this clone.

### How does cassiicolin manipulate immunity?

Plants have evolved two levels of immunity. The perception of microbe-derived or damage-derived molecular patterns (MAMPs/DAMPs) by the PRR membrane receptors triggers innate immunity known as PTI (pattern-triggered immunity). The second level of immunity involves the perception of specific effectors, secreted by the pathogen, by specific intracellular NLR plant receptors, resulting in a hypersensitive response, with various outcomes depending on the trophic mode of the pathogen. More precisely, effectors may have a dual function [46]. Their original role may be to manipulate the basal immune defenses (PTI) in order to allow easier penetration and colonization by the pathogen. In addition, they may operate as triggers of the hypersensitive response, but only in hosts carrying cognate NLR receptors able to specifically interact with them.

Our study, describing the transcriptomic response induced by the purified effector cassiicolin in susceptible and tolerant rubber clones, yielded two striking results: 1) cassiicolin induced late but huge transcriptional reprogramming in the susceptible clone PB260, characterized by the massive induction of phosphorylation events, signature of intense signalization cascades; 2) comparatively, the transcriptional response in the tolerant clone RRIM600 was moderate, occurring earlier but stable over time, with classical features of defense reactions also found in the susceptible clone. These results are discussed below.

#### Cassiicolin and PTI

Cassiicolin is not a PAMP and therefore not expected to induce PTI. Indeed, PAMPs are generic molecules shared by a wide range of genera within the same kingdom, while cassiicolin is highly specific for the genus *Corynespora* [7]. It was purified to homogeneity [3] so that the presence of contaminating fungal PAMPs can be ruled out. However, it was applied after gentle abrasion of the lower epidermis, to allow penetration into the cells, which likely induced DAMP-triggered immunity (DTI), similar in every way to PAMP-triggered immunity [47]. To characterize the cassiicolin effect independently of wounding, DEGs were identified against wounded reference samples (abraded leaves without cassiicolin) having also likely developed DTI. Our results revealed that cassiicolin induced significant modifications of defense-associated pathways such as cell recognition, oxidoreduction processes or secondary metabolism, in both clones. The ‘cell recognition’ cluster included a high number of DEGs encoding PRRs, the PAMP/DAMP sensors at the origin of PTI (Table 3). Among those, LRR receptor-like serine/threonine-protein kinases were highly represented, including the FLS2 and EFR involved in the perception of the bacterial PAMPs Flagellin and EF-Tu respectively [48, 49]. In the same category, SOBIR1 can promote activation of plant defenses and cell death upon bacterial infection in *A*. *thaliana* [50]. In our study this gene was up-regulated in PB260 (log2 fold change of 5.14, S3 Table) but not differentially expressed in RRIM600. Receptor kinases containing lectin domains were also highly represented in our study. Some of these are involved in plant resistance towards fungal pathogens such as *Phytophtora infestans* [51] or *Botrytis cinerea* [52]. Out of the only three PRRs differentially expressed at 12 hpt in PB260, one was a wall-associated kinase (WAK, S3 Table). In *A*. *thaliana*, up-regulation of WAK1 enhanced resistance toward the necrotrophic fungus *B*. *cinerea* [53]. Cysteine-rich receptor-like kinases (CRKs) play important roles in the regulation of defenses against pathogens and programmed cell death, in *A*. *thaliana* [54]. PRRs containing a LysM domain, such as CERK1 (chitin elicitor receptor kinase 1) are activated by chitin in *Arabidopsis thaliana* [55].

Although cassiicolin induced transcriptional modifications related to PTI in both clones, these responses occurred later in the susceptible clone compared to the tolerant clone, as illustrated by the low number of up-regulated PRRs in PB260-12h compared to RRIM600-12h, or the enriched ‘defense response’ supercluster evidenced at 12 hpt in RRIM600 only. It is unclear whether cassiicolin manipulates PTI directly or indirectly, by modulating DTI. The variations at PTI level may not be sufficient to explain the difference in susceptibility between the two clones, which suggests that another mechanism is involved.

#### Cassiicolin and NETS

Massive transcriptional reprogramming in the susceptible clone PB260, with more than 20,000 DEGs 24 hours after cassiicolin Cas1 application, against less than 4,000 in the tolerant clone RRIM600 (Fig 1), is in keeping with the hypothesis that cassiicolin is indeed a necrotrophic effector able to trigger a hypersensitive response, as described in other necrotrophic pathosystems. In susceptible wheat cultivars, massive transcriptional reprogramming was observed after treatment with the necrotrophic effectors ToxA or Tox B from *Pyrenophora tritici-repentis* [27, 56]. Both toxins were able to activate defense-related pathways, together with ROS accumulation and impairment of photosynthesis, suggesting that they may take advantage of the HR defense system to trigger cell death. However, these studies did not compare the responses in susceptible and tolerant cultivars. ToxA interacts with an NLR gene conferring susceptibility [57], thus complying with the inverse gene-for-gene (NETS) model characteristic of necrotrophic interactions [58]. In the wheat / *Stagonospora nodurum* necrotrophic pathosystem, five secreted fungal effectors (SnToxA, SnTox1, SnTox2, SnTox3 and SnTox4) interact with five susceptibility factors (Tsn1, Snn1, Snn2, Snn3 and Snn4) respectively, inducing a hypersensitive response, which leads to disease [59–61]. The susceptibility genes characterized so far (Tsn1, Snn1 and Snn3) all belong to the NLR family.

Thanks to the re-annotation of our synthetic transcriptome (Blast2GO, Table 2), we were able to highlight the specific enrichment in DEGs associated with phosphorylation, in the susceptible clone PB260, suggesting triggering of a massive signalization cascade in response to cassiicolin. Such a signalization cascade is a feature common to both PTI and ETI [62]. However, while PTI and ETI responses are qualitatively similar, they differ in their intensity, with ETI responses more prolonged and robust than those in PTI [63, 64].

Owing to the similitudes of our results with the NETS response described in other necrotrophic pathosystems, we may anticipate that cassiicolin putatively interacts with a specific NLR protein to trigger a hypersensitive response. We have identified putative NLR transcripts in our synthetic transcriptome, among which 9% were significantly regulated in response to cassiicolin treatment, mostly in PB260 (Table 3). TMV resistance protein N was the most highly represented category among the TIR-NLR family. It was followed by the RML1B-like receptors found to confer resistance to the hemibiotrophic fungus *Leptosphaeria maculans* in *A*. *thaliana* [65] The most significantly up-regulated NLR (e-value of 8,54E-67) found in our study belongs to the RPP8 category, in the CC-NLR family (S3 Table). In *A*. *thaliana*, LOV1, receptor-like protein of the RPP8 category, mediates sensitivity to victorin, a toxin secreted by the necrotrophic fungus *Cochliobolus victoriae* [66]. In the absence of LOV1, victorin inhibits thioredoxin TRX-h5, which results in compromised defense but no disease symptoms. In the presence of LOV1, victorin binding to thioredoxin activates LOV1 and induces a hypersensitive response conferring sensitivity to *C*. *victoriae*. In 2015, a gene of the CC-NLR family was identified by fine-mapping of the cca-3 locus associated with resistance to *C*. *cassiicola* in cucumber [67]. The highly represented RGAs (resistance gene analogs) are putative immune receptors with NLR structure [68]. One member of the RGA3 category was among the rare transcripts with opposite transcription profiles between the two clones (Fig 2). It was found down-regulated in RRIM600 and up-regulated in PB260 (Hb_076771_010, S3 Table), although with low log2 fold change values (1.18 in PB260 and -1.42 in RRIM600, at 24 hpt).

Immune responses need to be tightly regulated to keep the balance between beneficial and detrimental effects for the plant. Fine tuning of the immune receptors gene expression is one of the mechanisms allowing the regulation of the hypersensitive responses [69].

## Conclusion

Our study presents the whole transcriptome profiling of susceptible and tolerant rubber tree clones in response to purified effector Cas1, secreted by the necrotrophic fungus *C*. *cassiicola*. Both clones activated basal defense responses via redox signaling, production of pathogenesis-related protein or secondary metabolism. However, the susceptible PB260 clone induced delayed but much stronger transcriptional reprograming than the tolerant clone, with all the features of a hypersensitive response (HR). This HR leading to cell death confers resistance against biotrophic pathogens (ETI) but leads to the disease in necrotrophic pathosystems (NETS). These results encourage us to search for a putative immunereceptor protein expected to interact with cassiicolin, whether directly or not, to induce this HR. By eliminating clones carrying such susceptibility factors from planting recommendations and selection programs, one can hope to reduce the pathogen pressure and thus limit CLF disease.

## Supporting information

S1 Table Full functional annotation of the rubber tree synthetic transcriptome. https://figshare.com/s/3a1503caa60b839ab905. Fasta sequences of the synthetic transcriptome are also available as a single file *via* https://doi.org/10.6084/m9.figshare.14565426.v1.
(PDF)

S2 Table RNA-Seq metrics. (XLSX)

S3 Table Genes differentially expressed between cassiicolin-treated and untreated leaves, in susceptible (PB260) and tolerant (RRIM600) rubber tree clones, 12 and 24 hours after treatment. (XLSX)

S4 Table Significantly enriched GO terms associated with DEGs in the four conditions: PB260-12h, PB260-24h, RRIM600-12h and RRIM600-24h. In each condition, up- and down-regulated transcripts were analyzed separately (only up-regulated for PB260-12h) and sorted according to the three GO categories: molecular function, biological process and cellular component. The REVIGO program was used to estimate relative proportions of non-redundant GO terms in each category, expressed as percentage of genes annotated with the term. Superclusters were indicated when several GO terms were loosely related according to REVIGO.
(XLSX)

S1 Fig Principal component analysis (PCA) of normalized RNA-Seq data for the PB260 (A) and RRIM600 (B) samples. PCA were performed with regularized log-transformed (rld) read counts. The plots were generated using the DESeq2 package and customized with the ggplot2 package. The samples are spanned in two-dimensional plan by their first two principal components (PCA1 and PCA2). Axis percentages indicate variance contribution. Colors discriminate the samples according to the applied treatment: abraded control (AC) in red and purified toxin (TOX) in blue. Shapes discriminate the samples according to treatment duration (hpt, for hours post-treatment): circles for 12 hpt and triangles for 24 hpt.
(TIF)

S2 Fig Hierarchical clustering of PB260 sample-to-sample distances, 12h (A) and 24h (B) after cassiicolin treatment. Heatmap showing the Euclidean sample-to-sample distances between regularized log transformed values, calculated with the DESeq2 package and using the heatmap.2 function from the gplots package. Colors illustrate the distances, from the shortest (dark) to the longest (light). Sample names are indicated below the heatmap, as described in S2 Table. Treatments are specified on the side: AC, Abraded Control and TOX, purified toxin.
(TIF)

S3 Fig Hierarchical clustering of RRIM600 sample-to-sample distances, 12 h (A) and 24 h (B) after cassiicolin treatment. Heatmap showing the Euclidean sample-to-sample distances between regularized log transformed values, calculated with the DESeq2 package and using the heatmap.2 function from the gplots package. Colors illustrate the distances, from the shortest (dark) to the longest (light). Sample names are indicated below the heatmap, as described in S2 Table. Treatments are specified on the side: AC, Abraded Control and TOX, purified toxin.
(TIF)

S4 Fig MA-plots showing the distribution of DEGs induced by cassiicolin treatment in PB260 and RRIM600 samples at 12 and 24 hours post-treatment. The four conditions (PB260-12h, PB260-24h, RRIM600-12h and RRIM600-24h) were analyzed independently. Plots were generated using the plotMA function from the DESeq2 package. Each dot represents a gene, not differentially (grey) or differentially expressed (red), in cassiicolin-treated samples compared to the untreated controls, with a p-value threshold of 0.1. For each gene, the log2 fold changes are plotted on the y-axis and the mean of normalized count are plotted on the x-axis.
(TIF)

S5 Fig REVIGO treemap representation summarizing GO molecular function categories over-represented in PB260 (A and C) and RRIM600 (B and D) up-regulated genes. Treemaps were performed separately for 12 hpt (A and B) and 24 hpt (C and D). They were built taking the percentages of genes annotated with the non-redundant GO terms from S4 Table (REVIGO treemap % column). Each rectangle represents a significant GO term, sometimes joined into colorized superclusters of loosely related terms, whose names are labelled in white squares. The size of the rectangles is proportional to the p-value given by Blast2GO analysis (i.e. the larger the rectangle, the more significant the GO-term).
(TIF)

S6 Fig REVIGO treemap representation summarizing GO molecular function categories over-represented in RRIM600 (A and C) and PB260 (B) down-regulated genes. Treemaps were performed separately for 12 hpt (A) and 24 hpt (B and C). They were built taking the percentages of genes annotated with the non-redundant GO terms from S4 Table (REVIGO treemap % column). Each rectangle represents a significant GO term, sometimes joined into colorized superclusters of loosely related terms, whose names are labelled in white squares. The size of the rectangles is proportional to the p-value given by Blast2GO analysis (i.e. the larger the rectangle, the more significant the GO-term).
(TIF)
