# Supplementary material for: Transcriptome profiling in susceptible and tolerant rubber tree clones in response to cassiicolin Cas1, a necrotrophic effector from Corynespora cassiicola
Source: PLoS One. 2021 Jul 28;16(7):e0254541. doi: 10.1371/journal.pone.0254541 (PMC8318233; doi:10.1371/journal.pone.0254541)
Supplement: S1 Table — https://figshare.com/s/3a1503caa60b839ab905. Fasta sequences of the synthetic transcriptome are also available as a single file via https://doi.org/10.6084/m9.figshare.14565426.v1. (PDF) [file pone.0254541.s001.pdf]

S1 Table. Full functional annotation of the rubber tree synthetic transcriptome. https://figshare.com/s/3a1503caa60b839ab905. Fasta sequences of the synthetic transcriptome are also available as a single file *via* https://doi.org/10.6084/m9.figshare.14565426.v1
