# Supplementary figures and images for: Transcriptome profiling in susceptible and tolerant rubber tree clones in response to cassiicolin Cas1, a necrotrophic effector from Corynespora cassiicola
Source: PLoS One. 2021 Jul 28;16(7):e0254541. doi: 10.1371/journal.pone.0254541 (PMC8318233; doi:10.1371/journal.pone.0254541)

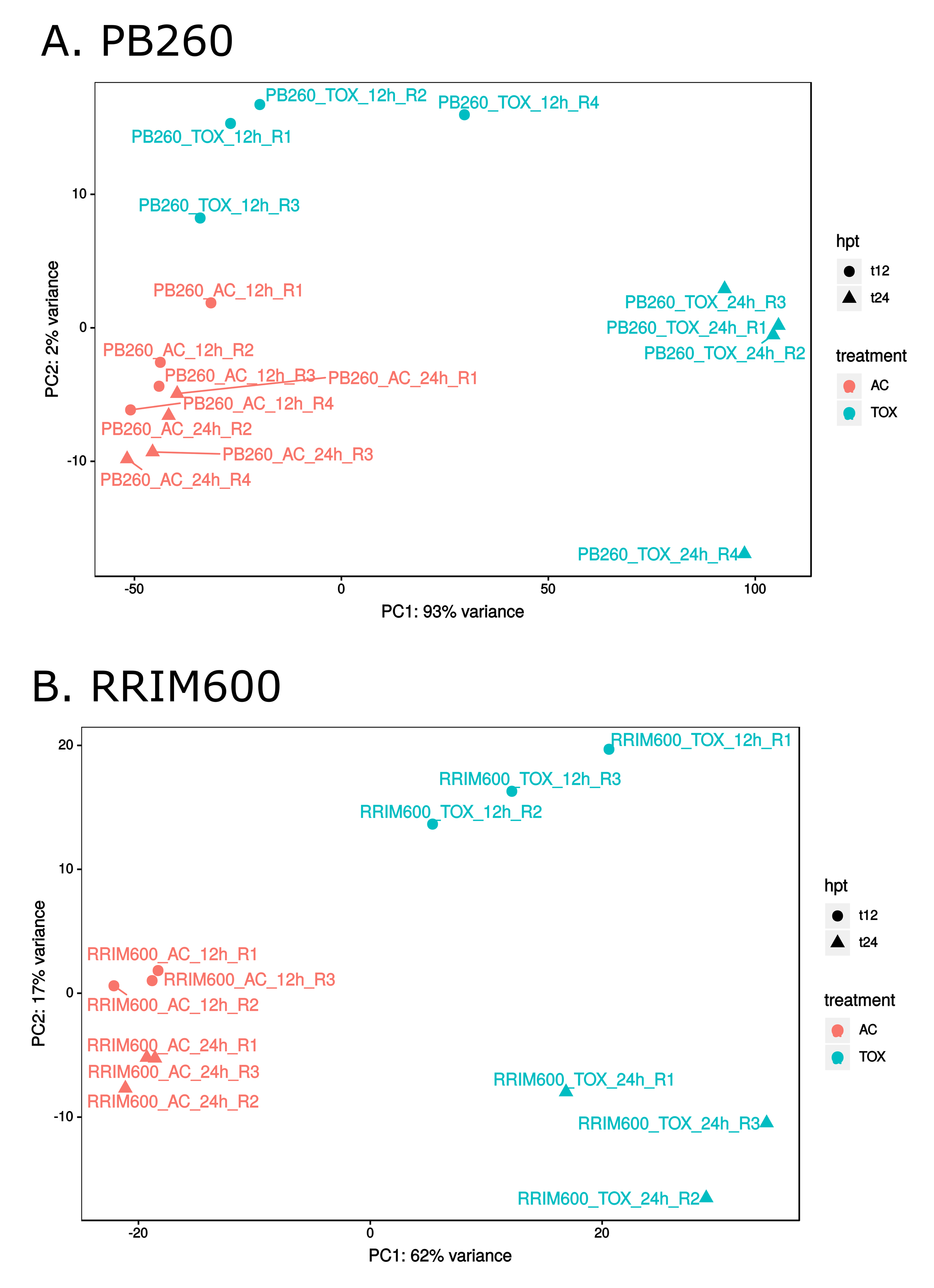

Supplement: S1 Fig — PCA were performed with regularized log-transformed (rld) read counts. The plots were generated using the DESeq2 package and customized with the ggplot2 package. The samples are spanned in two-dimensional plan by their first two principal components (PCA1 and PCA2). Axis percentages indicate variance contribution. Colors discriminate the samples according to the applied treatment: abraded control (AC) in red and purified toxin (TOX) in blue. Shapes discriminate the samples according to treatment duration (hpt, for hours post-treatment): circles for 12 hpt and triangles for 24 hpt. (TIF) [file pone.0254541.s005.tif]

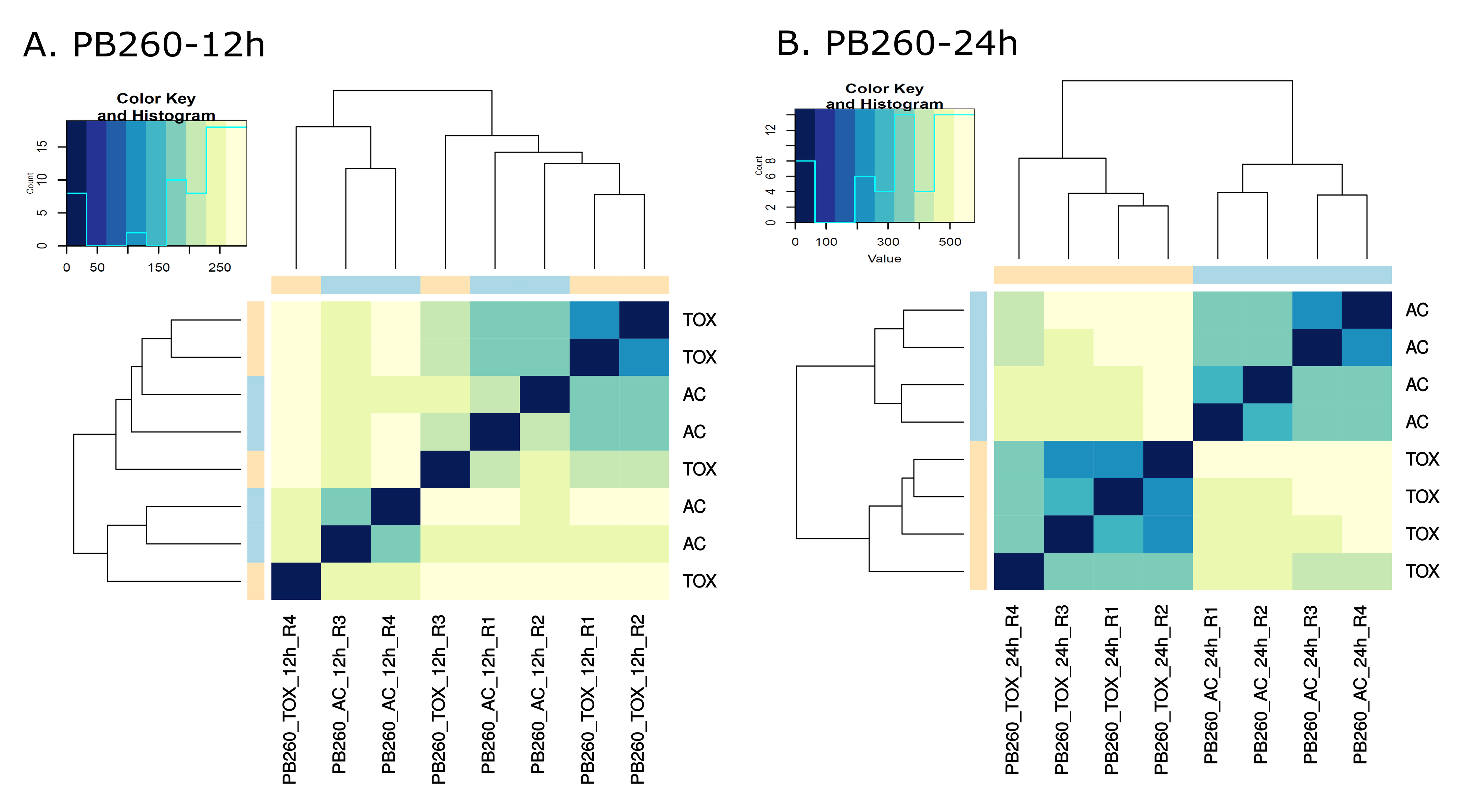

Supplement: S2 Fig — Heatmap showing the Euclidean sample-to-sample distances between regularized log transformed values, calculated with the DESeq2 package and using the heatmap.2 function from the gplots package. Colors illustrate the distances, from the shortest (dark) to the longest (light). Sample names are indicated below the heatmap, as described in S2 Table. Treatments are specified on the side: AC, Abraded Control and TOX, purified toxin. (TIF) [file pone.0254541.s006.tif]

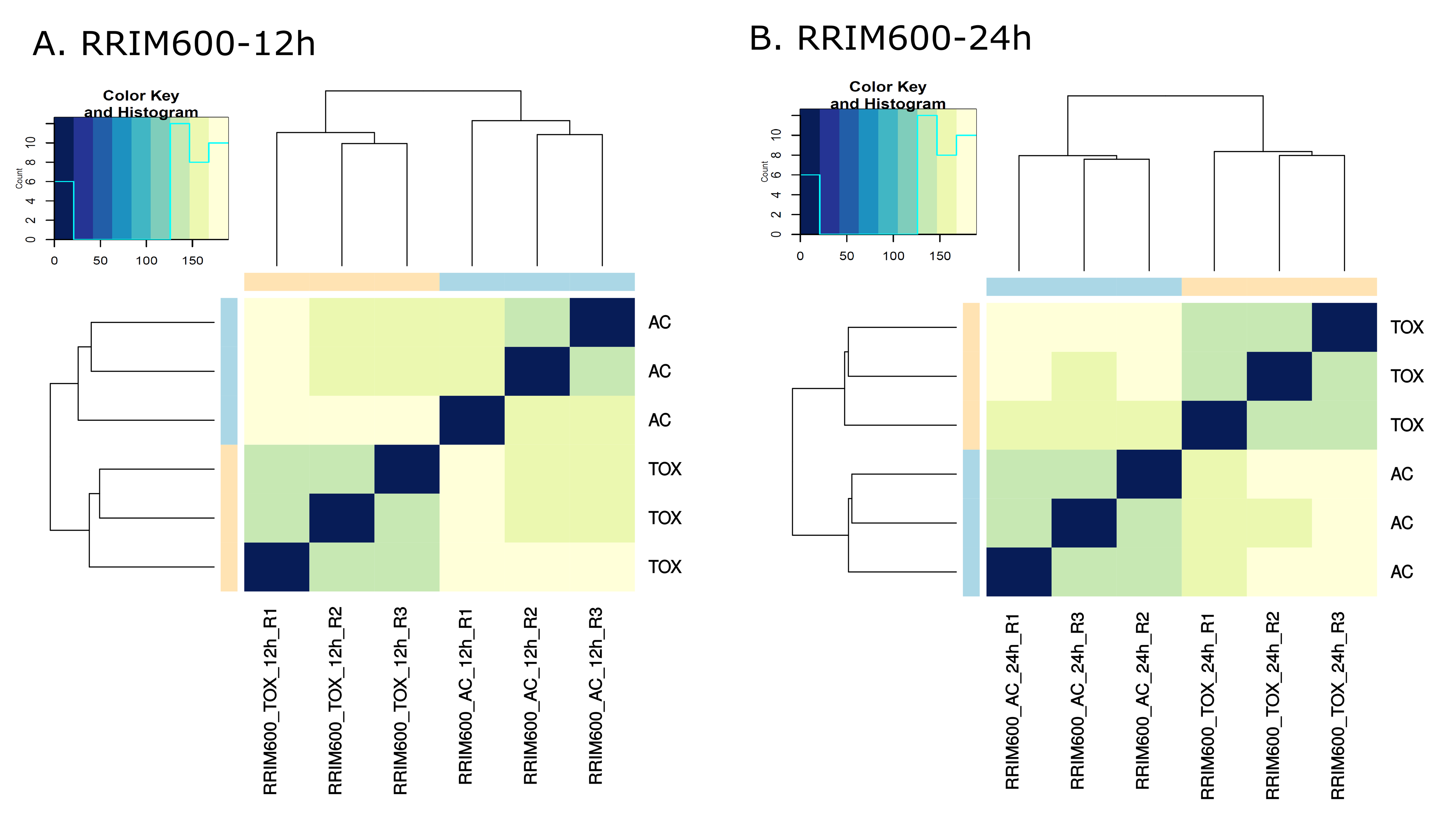

Supplement: S3 Fig — Heatmap showing the Euclidean sample-to-sample distances between regularized log transformed values, calculated with the DESeq2 package and using the heatmap.2 function from the gplots package. Colors illustrate the distances, from the shortest (dark) to the longest (light). Sample names are indicated below the heatmap, as described in S2 Table. Treatments are specified on the side: AC, Abraded Control and TOX, purified toxin. (TIF) [file pone.0254541.s007.tif]

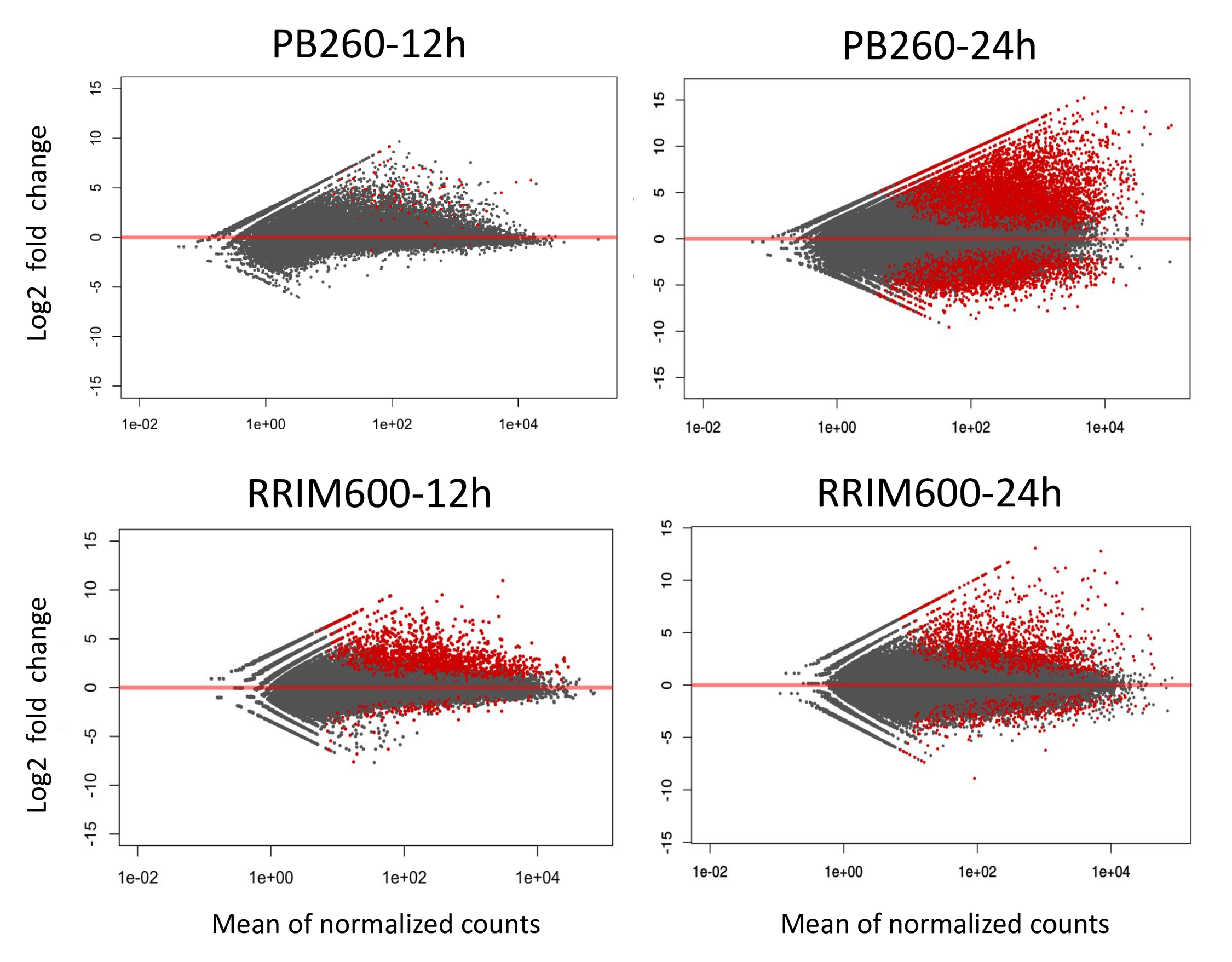

Supplement: S4 Fig — The four conditions (PB260-12h, PB260-24h, RRIM600-12h and RRIM600-24h) were analyzed independently. Plots were generated using the plotMA function from the DESeq2 package. Each dot represents a gene, not differentially (grey) or differentially expressed (red), in cassiicolin-treated samples compared to the untreated controls, with a p-value threshold of 0.1. For each gene, the log2 fold changes are plotted on the y-axis and the mean of normalized count are plotted on the x-axis. (TIF) [file pone.0254541.s008.tif]

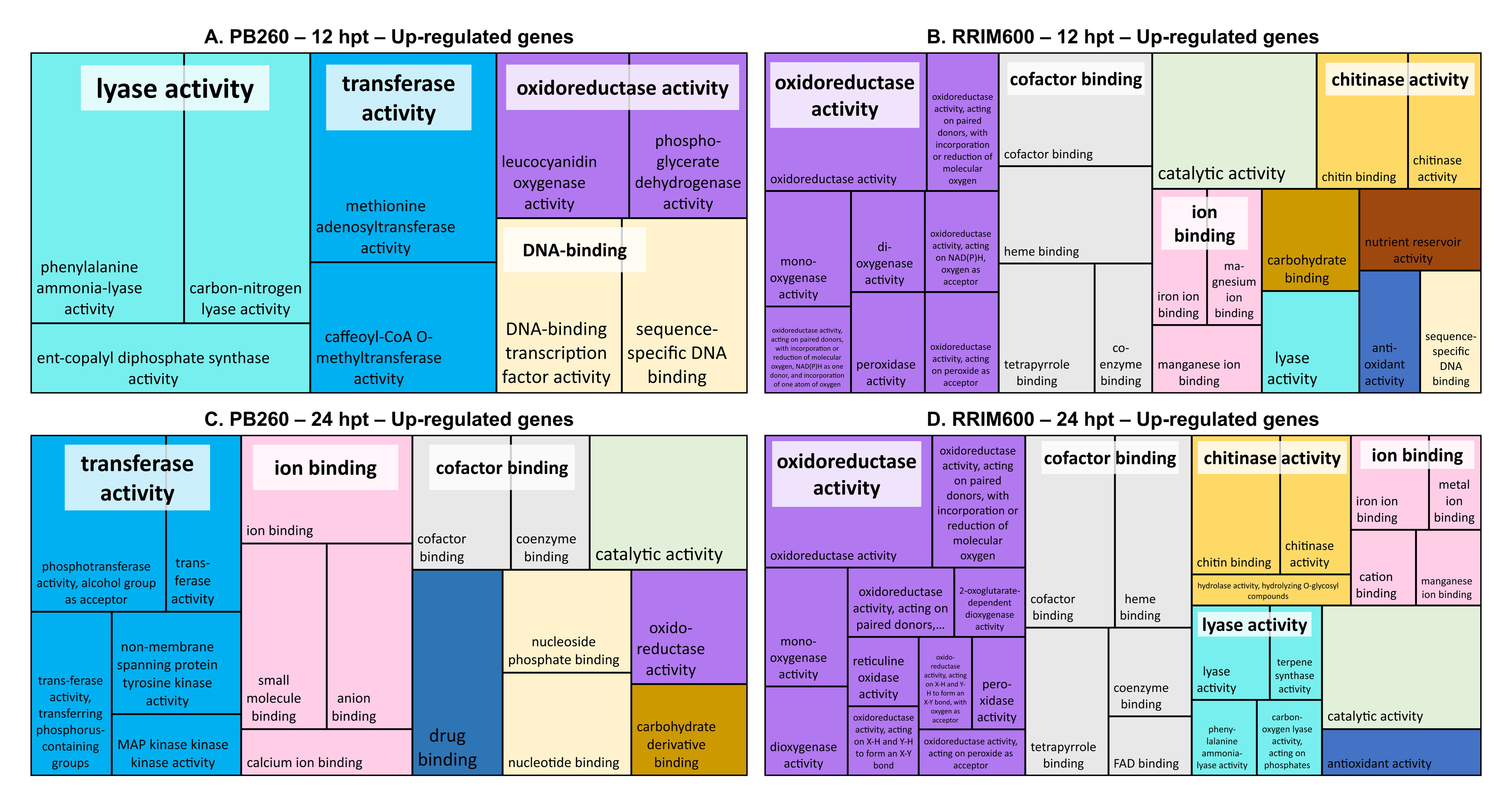

Supplement: S5 Fig — Treemaps were performed separately for 12 hpt (A and B) and 24 hpt (C and D). They were built taking the percentages of genes annotated with the non-redundant GO terms from S4 Table (REVIGO treemap % column). Each rectangle represents a significant GO term, sometimes joined into colorized superclusters of loosely related terms, whose names are labelled in white squares. The size of the rectangles is proportional to the p-value given by Blast2GO analysis (i.e. the larger the rectangle, the more significant the GO-term). (TIF) [file pone.0254541.s009.tif]

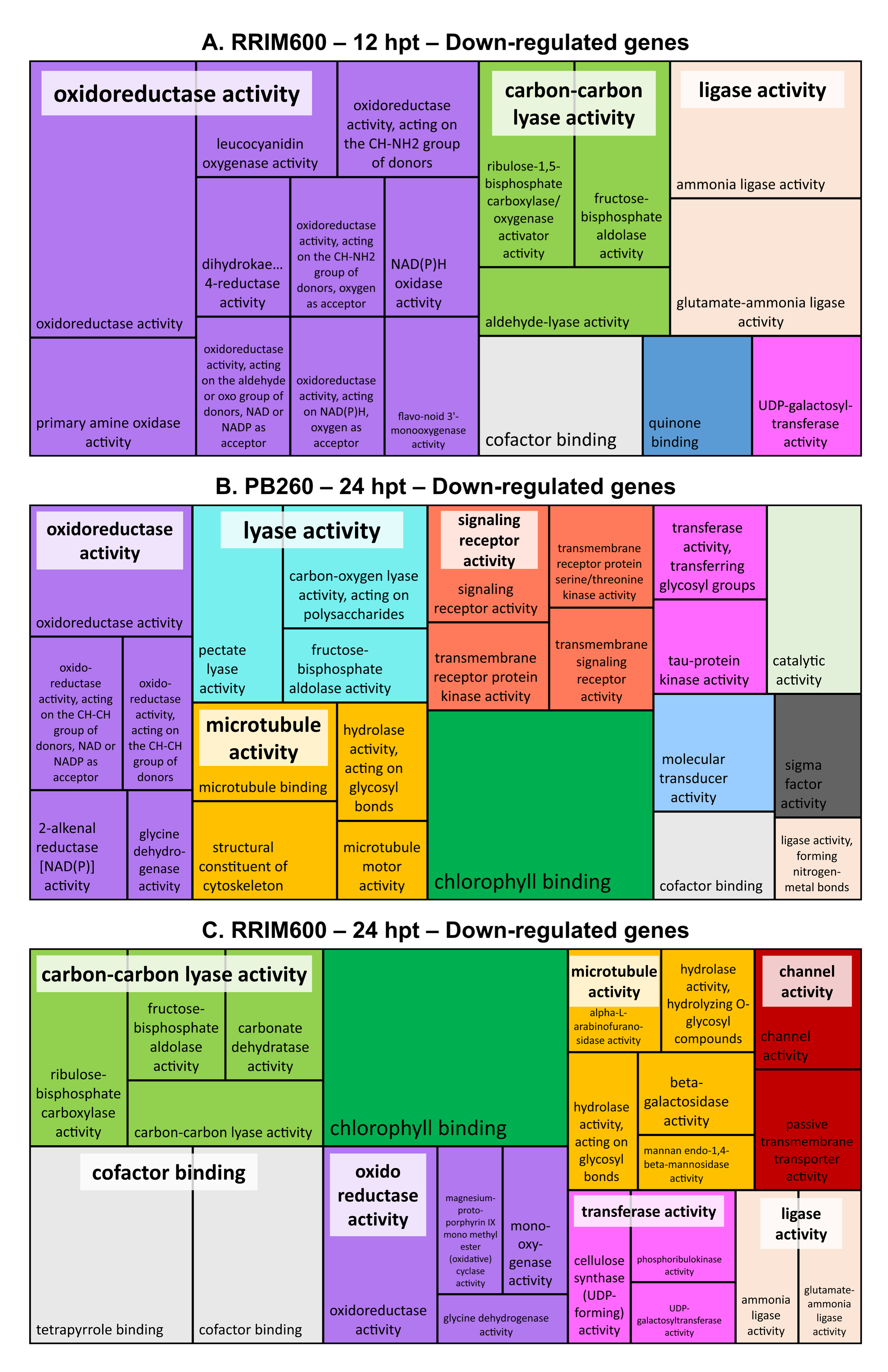

Supplement: S6 Fig — Treemaps were performed separately for 12 hpt (A) and 24 hpt (B and C). They were built taking the percentages of genes annotated with the non-redundant GO terms from S4 Table (REVIGO treemap % column). Each rectangle represents a significant GO term, sometimes joined into colorized superclusters of loosely related terms, whose names are labelled in white squares. The size of the rectangles is proportional to the p-value given by Blast2GO analysis (i.e. the larger the rectangle, the more significant the GO-term). (TIF) [file pone.0254541.s010.tif]
